# Investigating the influence of perinatal nicotine exposure on genetic profiles of neurons in the sub-regions of the VTA

**DOI:** 10.1038/s41598-020-59248-0

**Published:** 2020-02-12

**Authors:** Tina Kazemi, Naze G. Avci, Renee F. Keller, Yasemin M. Akay, Metin Akay

**Affiliations:** University of Houston, Department of Biomedical Engineering, Houston, TX 77204 USA

**Keywords:** Development, Neurological disorders

## Abstract

Chronic nicotine exposure during pregnancy has been shown to induce physiological and anatomical alterations in offspring. Previously, we investigated the complexity of dopamine (DA) neuron firing in the sub-regions of the ventral tegmental area (VTA) following perinatal nicotine exposure. Using approximate entropy, we found that within the middle sub-region, the parainterfascicular nucleus (PIF), there was higher complexity indicating more random neural firing and a less homogeneous neuron population. Therefore, we sought to investigate the neuron populations within the sub-regions of the VTA following perinatal nicotine exposure. We used real time PCR in order to find the relative quantity of glutamate to γ-aminobutyric acid (GABA), DA, and glutamate neurons within three sub-regions: the parabrachial pigmented nucleus (PBP), parainterfascicular nucleus (PIF), and paranigral nucleus (PN). Our results showed that the PIF region of the VTA contained a more diverse population of neurons resulting in a more complex system. In addition, we found that DA neurons are more activated in PN sub-region of the VTA, which mediates the rewarding effects of drugs including nicotine. Lastly, using immunohistochemistry, we observed an overall decrease in DA neurons following perinatal nicotine exposure.

## Introduction

Maternal smoking during pregnancy is a major public health concern with women who continue smoking throughout their pregnancies^1^. As a result, 40% of children in the world are estimated to be perinatally exposed to maternal smoking^2^. Maternal smoking has been implicated as a risk factor for developmental and cognitive disorders in the offspring resulting in low birth weight^3^, increased risk of stillbirth^4^, sudden infant death syndrome (SIDS)^5^, predisposition towards nicotine dependence and substance abuse^6,7^, attention deficit hyperactivity disorder (ADHD)^8^, as well as many neurocognitive deficits^9,10^.

Although tobacco contains over four thousand chemicals, nicotine has been identified as the biologically active and most addictive substance in tobacco^11^. Prenatal nicotine studies have shown that exposure to nicotine alters the programming of neurodevelopmental events on a cellular level^9^, which causes structural changes in the nervous system, including the migration of nerve cells, synapse function, and localization of specific nerve cell populations as well as alterations to axons, dendrite, spines and specific regions of the CNS^12–15^. Furthermore, nicotine activates dopaminergic (DA) neurons of the mesocorticolimbic pathway also known as the reward circuitry of the brain to initiate dependence^16^. In the mesocorticolimbic pathway, DA neurons originating from the ventral tegmental area (VTA) project to the striatum, prefrontal cortex (PFC), and nucleus accumbens (NAc). Exposure to nicotine initiated by the activation of VTA promotes reward-driven behavior and produces reinforcing effects associated with drug exposure^17^. Moreover, offspring are exposed to nicotine, which readily crosses the placenta during gestation, but also subsequent to birth through breast-feeding, as breast milk contains nicotine concentrations that are two to three times higher than the mother’s plasma concentrations^18,19^.

Previously, we investigated the functional coupling between the PFC and the VTA in order to determine how the disruption in communication affects neuronal firing patterns of the VTA DA neurons^20^. Our results revealed that nicotine exposure triggers VTA DA neuronal firing significantly when communication between the PFC and VTA was intact, while the effects of exposure on VTA DA neuron firing were eliminated once the connection between PFC and VTA was transected. From these results, we later investigated the dynamics of DA neurons within sub-regions of the VTA using *in vivo* electrophysiological recordings to characterize and quantify the dynamics of neural activity in three main VTA regions: the parabrachial pigmented nucleus (PBP), the parainterfascicular nucleus (PIF) and the paranigral nucleus (PN). Approximate entropy (ApEn) was used to measure the regularity of neural activities, where higher values are indicative of a more complex system and reduced regulatory system. We found maternal nicotine exposure affected the complexity of neuronal firing in the DA neurons in a non-uniform manner in the VTA sub-regions^21^. Our results showed higher complexity in the PIF sub-region among the experimental group specifically, more random neuronal firing from a population of neurons with decreased homogeneity. The saline-treated control group, however, exhibited lower complexity, which may be the result of synchronous activity from a homogenous population of neurons. The complexity values in the mentioned study were correlated to neuron homogeneity such that a higher ApEn value indicated increased neural activities, while a lower ApEn value indicated decreased neural activities^21^. Other studies have also determined that perinatal nicotine exposure affect dopamine release as well as dopamine levels and turnover in the fetal forebrain depending on the specific region of the dopaminergic system^22–24^. It has been revealed that after the nicotine injection, some VTA DA neurons showed different temporal changes on the firing rate. Altogether, these result suggested that detailed subgroups of VTA DA may exist within VTA, defined by physical function and/or anatomical sub-region^25–28^ but several studies suggested that the posterior VTA, but not anterior VTA, centering around the PN mediates the rewarding effects of drugs including nicotine^28^. However, more information regarding the DA existence in different sub- regions of VTA investigating the effects of developmental nicotine exposure is needed to better understand potential targets for novel medical treatments. Therefore, in this current study, we investigated the nicotine-dependent activation of specific neurons within the VTA sub-regions to understand the effect of perinatal nicotine exposure. To examine the population of neurons within the sub-regions of the VTA, we tested the specific gene expressions in rat neurons treated perinatally with nicotine. Real time PCR (RT-qPCR) was used to find the relative quantity of GABA, DA, and glutamate neuron markers and immunofluorescence staining was performed to visualize DA neurons based on their role in the reward/addiction pathway.

## Results

### Primer validation

**Figure 1:**
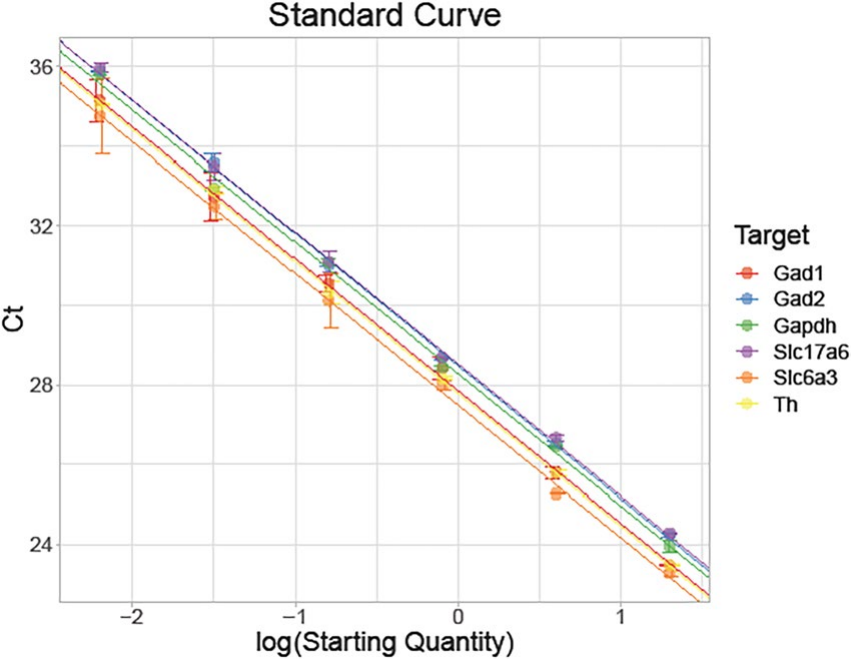
Validation of RT-qPCR to determine primer efficiency. To validate our primers, we conducted a standard curve experiment using 5x step dilution starting with 20 ng. Standard curves for the RT-qPCR primer validation were created by 5x step-wise dilution of total RNA from midbrain samples containing 20 ng to about 6 pg. The experiment was performed in triplicate. Data is presented as mean ± SEM. All primers passed the validation with acceptable efficiencies.

The VTA sub-regions (PBP, PIF, and PN) were isolated from rat pups (4 weeks old) that were perinatally exposed to nicotine or saline (control) through their mother via an osmotic pump as described in the methods section, in order to compare the genetic expressions between each sub-region of the VTA. Five candidate neuronal markers were selected based on their usage as neurotransmitters in gene expression studies and significance in neural mechanisms of addiction; Taqman Gene Expression Assays GAD1 and GAD2 playing role in the conversion of glutamate to GABA, the inhibitory neurotransmitter in central nervous systems, Slc6a3, Tyrosine Hydroxylase (TH) and Slc17a6 playing in dopamine transport and Slc17a6 (also known as VGluT2) playing role in L-glutamate transmembrane transporter activity in glutamatergic pathway. Standard curves for the RT-qPCR primer validation were created by 5x step-wise dilution of total RNA from midbrain samples containing 20 ng to about 6 pg (Fig. 1). Primer specificity was confirmed by melting curve analysis. All primers were given in Table 1.

**Table 1:** List of primer sequences for GABA, DA, and glutamate neurons.

Target Gene	Gene Symbol	Context Sequence	Category	Assay ID	Lot Number	Slope	Y-Intercept	R^2^	Eff%
Solute carrier family 6 (neurotransmitter transporter), member 3	Slc6a3	GTGGCCACAGATGGACCTGGGCTCA	Neurotransmitter transporter activity	Rn00562224_m1	1643391	−3.918	27.705	0.99878	100.02
Tyrosine hydroxylase	TH	CAAGGACAAGCTCAGGAACTATGCC	Molecular function unclassified	Rn00562500_m1	1613109	−3.703	28.013	0.99939	99.9
Solute carrier family 17 (vesicular glutamate transporter), member 6	Slc17a6	GGACAGATCTACAGGGTGCTGGAGA	Amino acid transmembrane transporter activity	Rn00584780_m1	1510464	−3.828	28.642	0.99742	100.02
Glutamate decarboxylase 1	Gad1	CAACCTGTTTGCTCAAGATCTGCTT	Carboxylyase activity	Rn00690300_m1	1670142	−3.766	28.108	0.99912	99.27
Glutamate decarboxylase 2	Gad2	CGATTAAAACAGGGCATCCCCGATA	Carboxylyase activity	Rn00561244_m1	1508579	−3.832	28.566	0.99912	99.91
Glyceraldehyde- 3-phosphate dehydrogenase	Gapdh	AGGAGTCCCCATCCCAACTCAGCCC	Housekeeping gene	Rn01775763_g1	1673786	−3.89	28.182	0.99742	100.07

### Comparison of the relative gene expression by VTA sub-region

**Figure 2:**
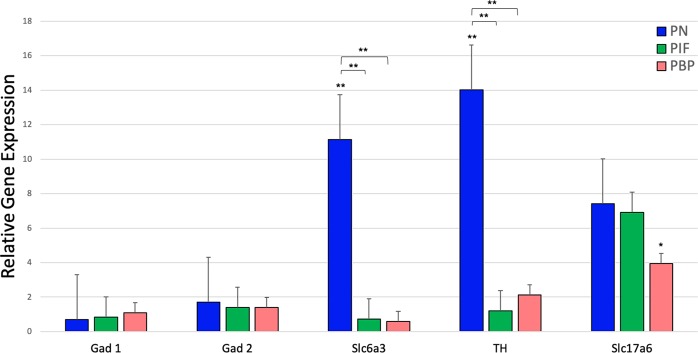
Relative gene expression of GABA, DA, and glutamate markers by VTA sub-region after the nicotine exposure in the rats. The results of quantitative RT-PCR validation experiments showed in the graph represent the fold change in the expressions of the selected genes relative to the expression of the housekeeping gene GAPDH. The red bars indicate the expressions of the selected genes in PBP, green bars indicate the expressions of the selected genes in PIF and blue bars indicate the expressions of the selected genes in PN. Two-way analysis of variance (ANOVA) was used to assess the difference between each gene within the sub-regions. Unpaired t-test was used when comparing gene expression profiles between nicotine and saline treatment groups in the sub-regions. *denotes p < 0.05, **denotes p < 0.01. Data is presented as mean ± SEM.

DA is a key regulator of reward behavior. To understand the DA neuron distribution and its effect on the dopaminergic pathway and within the reward circuitry in VTA midbrain, we compared nicotine-treated samples with saline-treated samples in three different VTA sub-regions. In the VTA, several genes important in regulating DA levels were measured. Within any of the sub-regions of VTA, the expressions of the GABA genes; GAD1 and GAD2 were not significantly different. There was a significantly higher expression of both DA genes, Slc6a3 and TH, in the PN sub-region compared to the PIF and PBP sub-regions of the VTA after gestational nicotine exposure. These markers did not show any significant changes when compared between the PIF and PBP sub-regions. The glutamate gene Slc17a6, which is also co-expressed in DA neurons did not show any significant expression in any parts of the VTA as shown in Fig. 2.

The expression of each gene within each sub-region was analyzed using Welch’s unpaired t-tests and results are summarized in Table 2. When compared to saline, GAD1 and GAD2 did not show any significance in any parts of VTA. Slc6a3 expression was significant in the PN region of VTA compared to both PIF and PBP sub-regions with p-values of 0.003 (t = 3.209, df = 22.18) and 0.002 (t = 3.664, df = 19), respectively (Table 2). When we compared TH expression in all three sub-regions, the expression in PN compared with PIF and PBP was statistically significant with p-values 0.006 (t = 3.253, df = 13.79) and 0.005 (t = 3.147, df = 23.96), respectively. However, there was no significant expression of TH when PIF sub- region was compared to PBP sub-region. Slc17a6 followed a similar pattern of expression compared to another DA markers that we used. However, we did not observe any significance within three sub-regions.

**Table 2:** RT-qPCR Gene Expression Summary and Statistics.

Sub-region	Relative Expression				
	GAD1	GAD2	Slc6a3	TH	Slc17a6
PN	0.706	1.71	11.14	14.03	7.41
PIF	0.84	1.39	0.71	1.19	6.90
PBP	1.10	1.39	0.58	2.14	3.94
	**p-values**				
	**GAD1**	**GAD2**	**Slc6a3**	**TH**	**Slc17a6**
PN vs. PBP	NS	NS	0.002	0.005	NS
PN vs. PIF	NS	NS	0.003	0.006	NS
PIF vs. PBP	NS	NS	NS	NS	NS

When we compared the expression of each gene in the specific VTA sub-regions with that of saline-treated control of the same sub-regions using a two-way ANOVA, we observed that Slc6a3 was significantly expressed in the PN sub-region of the nicotine- treated samples (F_2,4_ = 6.28, p = 0.006). TH expression was also significant in the PN sub-region of the nicotine-treated samples (F_2,4_ = 15.51, p = 0.0005). Finally, Slc17a6 expression was significantly higher in PBP sub-region of the nicotine-treated samples (F_2,4_ = 12.95, p = 0.036) (Fig. 2).

### Relative gene expression of TH in the VTA sub-regions compared to saline-treated whole midbrain VTA samples

**Figure 3:**
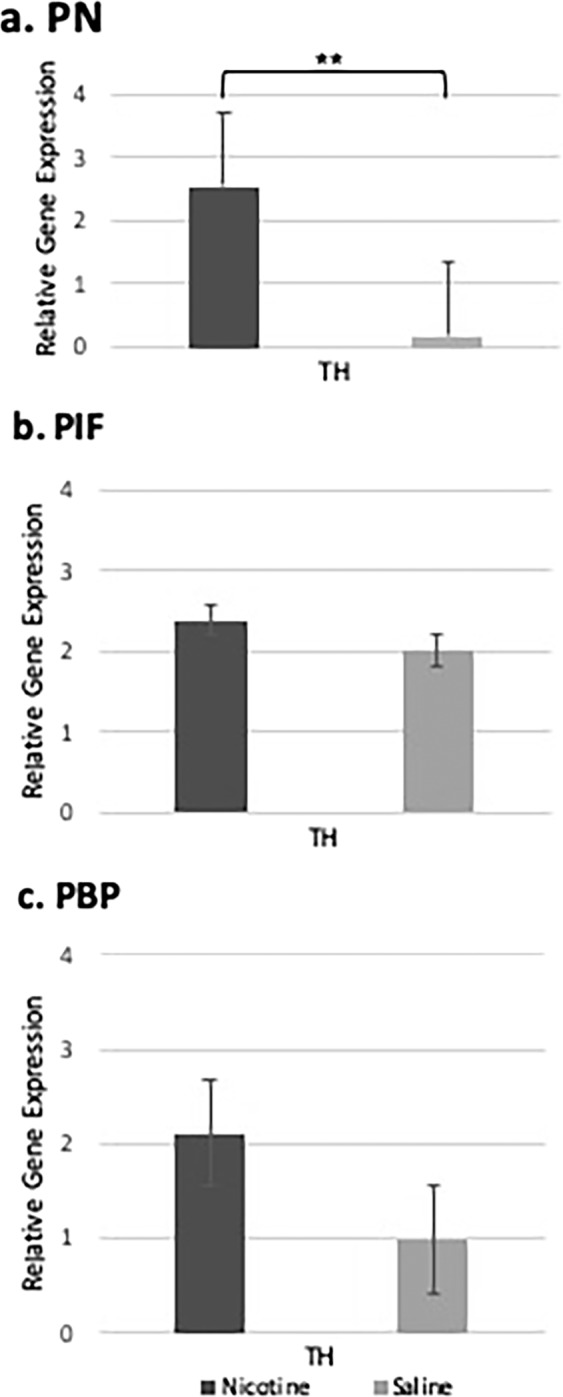
Relative gene expression of Th in the VTA sub-regions compared with the saline whole midbrain VTA samples. Using RT-qPCR, we compared the nicotine-treated and saline-treated Th, DA neurons’ expressions in three different sub-regions of the VTA midbrain with the saline-treated whole midbrain VTA in order to identify the DA neuron population per region. **(a)** Th expression in the PN sub-region of the VTA was significantly higher compared to saline-treated whole midbrain VTA samples. **(b**,**c)** No significant changes of Th expression were observed in the PIF and PBP sub-regions, respectively when we compared the Th expression with the saline whole midbrain VTA samples. **denotes p < 0.01. Data is presented as mean ± SEM.

We investigated dopamine neurons in the VTA using TH, which is the most well-known genetic marker for dopamine neurons and further investigated its expression in the sub-regions of VTA using RT-qPCR. Relative TH expression within each sub-region of nicotine and saline-treated samples were compared with the saline-treated whole midbrain VTA samples to identify higher neuron population per region using RT-qPCR (Fig. 3). As shown in Fig. 3a, we found that nicotine-treated samples had significantly higher expression of TH in the PN compared with saline-treated samples (t = 6.309, df = 5.99, p = 0.00074). Although in the PIF and PBP sub-regions, the expression of TH was slightly increased compared to saline-treated samples, there was no statistically significant difference in the TH expression following perinatal nicotine exposure (Fig. 3b,c).

### Immunohistochemistry staining of the nicotine and saline-treated VTA sub-regions

**Figure 4:**
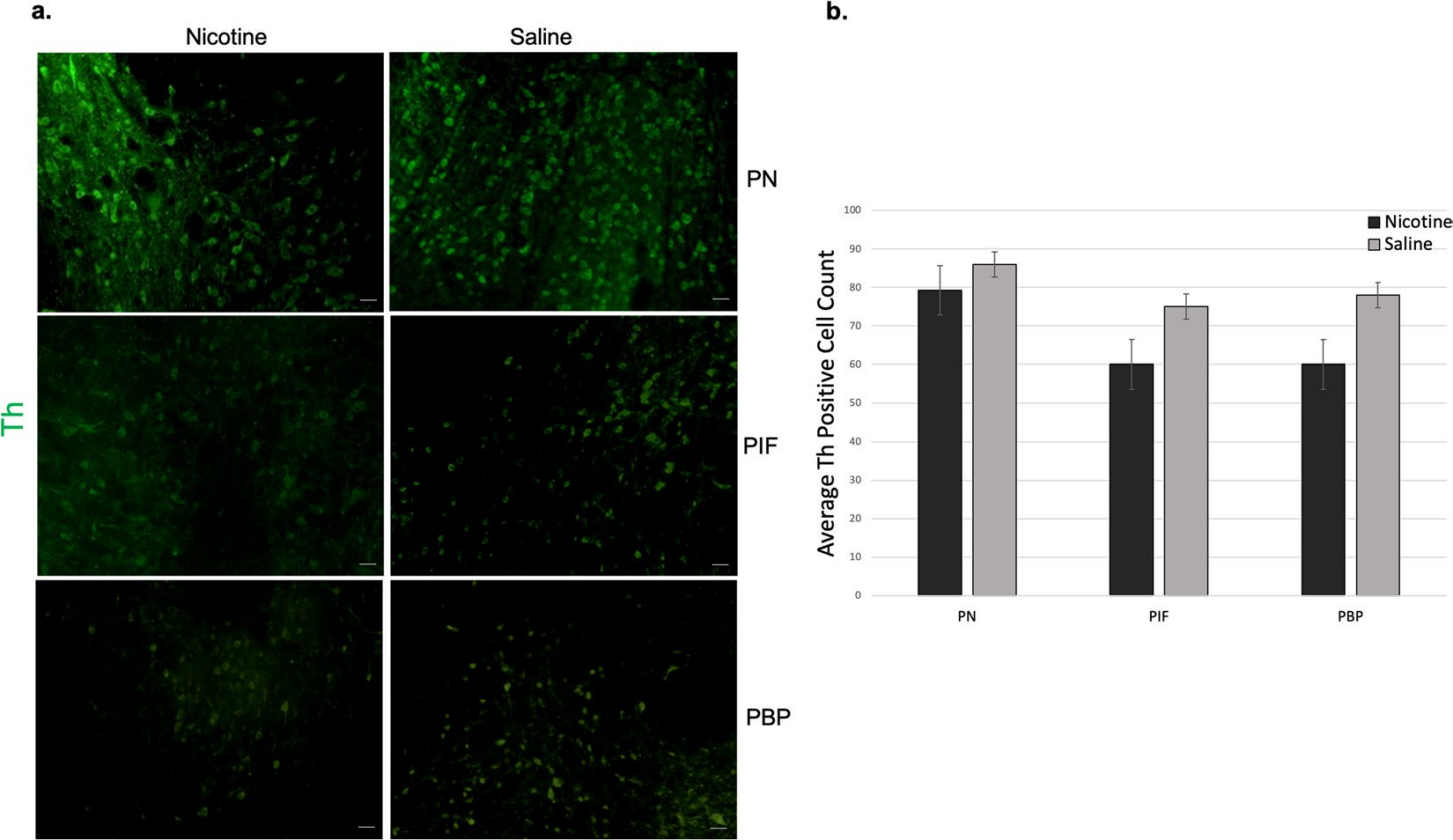
IF staining of Th in PN, PIF and PBP sub-regions of VTA midbrain. (**a**) Th is expressed by DA neurons in all VTA sub-regions. Freshly sliced nicotine and saline-treated samples were subjected to immunofluorescence staining to detect Th expression (green) in DA neurons. Scale bars represent 50 µm. **(b)** Average Th positive cell count based on IF staining. Although DA neuron numbers in saline-treated samples were higher, we did not observe any significance. Data is presented as mean ± SEM.

To characterize DA-positive cells, nicotine and saline-treated sub-region tissue samples were stained using immunofluorescence (IF) staining (Fig. 4). Freshly dissected sections from PN, PIF and PBP sub-regions of the VTA midbrain were labeled against TH antibody. In Fig. 4a, The IF results showed that both nicotine and saline- treated sections were positive for DA neurons. In the nicotine-treated samples, there was more DA neuron staining in the PN compared with the PBP and PIF sub-regions of the VTA due to perinatal nicotine exposure and after birth through breast milk with average cell count of 79, 69, and 70 respectively. Saline sections also showed a slightly higher DA staining in the PN sub-region of the VTA but overall more consistent DA staining within all sub-regions with average cell counts of 86 in PN, 74 in PIF, and 74 in PBP. Average TH positive cell counts were done for each sub-region and plotted as shown in Fig. 4b. Although, there was slightly higher DA neurons in saline-treated samples, it should be noted that the results are not statistically significant.

## Discussion

Perinatal nicotine exposure has been associated with altered burst firing of DA neurons^29^, decreased DA neurons in the VTA^26^, and altered DA release in response to nicotine administration^27^. Previously, we observed significant differences in neural firing between the sub-regions of the VTA indicating a more heterogeneous neuron population in the PIF sub-region caused by perinatal nicotine exposure. In the control case, more synchronous activity in response to perinatal nicotine exposure was evident, consistent with a more homogeneous neuron population^21^. This suggested that the PIF region of the VTA was more susceptible to nicotine exposure during neurodevelopment. Perinatal nicotine exposure followed by exposure to nicotine after birth through breast milk would provide important new data describing the effect of the maternal smoking on the brain’s reward and addiction system also highlighting the potentials for recovery. Therefore, we investigated the gene expression of neuronal markers within three VTA sub-regions in order to examine the different neuron populations (GABA, DA, and glutamate) and how they are modulated by perinatal nicotine exposure.

The sub-regions of the VTA have been described by several studies; however, we have focused our investigation on the VTA sub-regions that are DA cell-body-rich regions^28^. Moreover, subgroups of VTA DA neurons project to different areas of the brain and have been shown to elicit different responses to nicotine indicating different anatomical and physiological functions of VTA DA neurons^25^. Besides DA neurons, the VTA also contains GABA and glutamate neurons. We chose different markers for GABA (Gad1 and Gad2) and DA (Slc6a3, TH, Slc17a6) and one marker for glutamate (Slc17a6). Slc17a6 is an indication of glutamate expression with DA as it is expressed in DA neurons that co-express glutamate and DA^30,31^. Each of these markers have been used extensively to indicate the respective neurons. In order to quantify each neuron type, qPCR was used for its specificity. To test for the presence and relative amount of GABA neurons, we used Gad1 and Gad2, also known as Gad67 and Gad65, respectively, which are the most common GABA neuron markers^32,33^. Gad1 and Gad2 are glutamate decarboxylases, which are responsible for catalyzing glutamic acid into gamma-aminobutyric acid (GABA). Within the VTA, GABA neurons are responsible for inhibiting DA activation, and therefore, alteration in GABA neurons may have profound effects on DA firing. Our results suggested that GABA neurons were not significantly affected by nicotine exposure during development, based on the non-significant difference in relative expression between nicotine and saline-treated offspring. A study by Nair-Roberts investigated the total number of neurons in the VTA, SN, and RRF, and found large variation in the relative numbers of DA and GABAergic neurons between regions and within the regions. Within the VTA sub-regions, they consistently estimated more TH-positive neurons compared with Gad-positive neurons^33^. More specifically, they found a ratio of 2.01 TH/Gad mRNA-positive neurons in the PN, which is consistent with our results.

For DA neurons, we used the markers TH, Slc6a3 and Slc17a6. TH is the rate-limiting enzyme required to catalyze tyrosine to L-Dopa for DA synthesis and the most reliable indicator for DA neurons^34,35^. The solute carrier family 6 member 3 (Slc6a3), also known as dopamine transporter (DAT) is another widely used dopamine marker. Expression of the Vglut2/Slc17a6 gene encoding the Vesicular glutamate transporter 2 (VGLUT2) in midbrain DA neurons enables these neurons to co-release glutamate in the nucleus accumbens (NAc), an important regulation in drug addiction^36,37^. Glutamate neurons have most recently been identified using VGluT2^30^. To identify glutamate in our study, we used Slc17a6 (solute carrier family 17 member 6), a protein coding gene that encodes vesicular glutamate transporter 2 (VGluT2) and is the most common glutamate marker used in the midbrain^33,38,39^. A subset of glutamate VGluT2-positive neurons in the VTA are DA neurons, which co-release DA and glutamate to the NAc and results in increased bursting activity of DA neurons^30,40^. The co-release of DA and glutamate in the sub-population of VTA DA neurons is believed to play a crucial role in behavior activation induced by stimulants, playing a unique role in drug addiction^31,41^. Glutamate neurons were tested for using the Slc17a6 marker. The relative high expression of glutamate in our results suggests that perinatal nicotine exposure significantly affects the expression of Slc17a6, most likely in the subset of DA neurons that co-release DA and glutamate. These markers are extensively used for the detection of DA neurons^42^. Overall, we found significantly higher expression of DA markers TH and Slc6a3 in the PN compared with the saline control. An exception to this pattern was seen with Slc17a6, which was relatively high in PN compared to PIF and PBP sub-regions but did not show a statistical significance. It may be that not all TH-positive neurons express the Vglut2 gene after 6 weeks of birth. Mendez *et al*. showed that 25% of TH-positive DA neurons express the Vglut2 gene at birth, while only 14% keeps this expression after 6 weeks^43^. However, it should be noted that Slc17a6 expression was significantly higher in PBP sub-region of VTA when nicotine-treated samples were compared with saline-treated samples. This result is supported by the literature, where it has been shown that DA neurons expressing Vglut2 mRNA are most abundant in the PBP sub-regions of the VTA^44,45^. Our results concerning DA neurons suggest that perinatal nicotine exposure significantly affected DA neurons in the PN region. These results are consistent with the findings of Nair-Roberts *et al*., who reported a higher ratio of TH-positive neurons to Gad-positive neurons in the PN, indicating a larger quantity of DA neurons^33^.

Based on the RT-qPCR, we observed higher dopamine expression in the PN of nicotine-treated samples compared to the PN of those treated with saline. No significance was seen in the PIF and PBP regions of nicotine-treated samples compared to saline ones. Our IF staining results showed slightly higher DA neuron numbers in saline-treated samples; however, it should be noted that the results are not statistically significant. Dopamine expression within the PIF and PBP regions was not significantly changed between the nicotine and saline treated samples. The heterogeneity of midbrain DA neurons has been investigated in various research^46–48^. In fact, it is very likely that there are multiple distinct DA neuron subtypes within the sub-regions of VTA. Therefore, after perinatal nicotine exposure, the differences in the DA neuron expressions in the different sub-regions of VTA might be reflecting this heterogeneity, although we do not address the DA neuron diversity in this study.

In this study, we hypothesized that the nicotine exposure selectively affects the VTA sub-regions. In order to test this hypothesis, we used RT-qPCR and IF to investigate the DA neuron populations in the PN, PIF and PBP sub- regions of the VTA after perinatal nicotine exposure. We found that dopamine markers TH, and Slc6a3 to be significantly more expressed in the PN region of nicotine-treated samples when compared to PIF or PBP sub-regions within the same sample. Their expressions were also significantly higher in the nicotine-treated samples in PN sub-region when they were compared with the saline-treated samples of the same sub-region. Slc17a6 marker was significantly more expressed in PBP sub-regions of VTA when the nicotine-treated samples were compared to saline-treated samples however there was no significant difference in expression of nicotine-treated samples within the three sub-regions. We also investigated TH expression in the sub-regions of nicotine treated samples and saline treated samples compared with the whole VTA control sample treated with saline where we observed a significant expression of TH in the PN sub-region. No such significance was observed in TH expression among these samples in the PIF or the PBP sub-regions.

As a summary, this study aiming to define the long-term regulation of DA neurons in three different sub-regions of VTA showed that following perinatal nicotine exposure, DA neurons are more activated in PN sub-region of the VTA as suggested by Ikemoto *et al*.^28^. Identification of cell types and their locations are very important for determining the neuronal circuits and the interaction of neurons. Future studies should also investigate altered biological pathways using mRNA and miRNA expression profiles of DA and non-DA neurons following perinatal nicotine exposure in the VTA to further understand the addiction pathways and to develop potential drugs aiming to target this.

## Materials and Methods

### Animal treatment

All experimental protocols and surgical procedures were approved by the Institutional Animal Care and Use Committee (IACUC) and the University of Houston Animal Care Operations (ACO) and were performed in accordance with accepted guidelines and regulations. Pregnant female Sprague-Dawley (SD) rats (Charles River, Wilmington, MA, USA) were maintained on a 12-h light/12-h dark schedule at a temperature of 22 ± 2 °C and 65% humidity. Access to standard food and water was ad libitum. Rats were acclimated to the animal facility for 72 hours prior to receiving treatment on gestational day 7. An osmotic pump (Alzet, Cupertino, CA, USA) was implanted subcutaneously containing either nicotine hydrogen tartrate (Sigma-Aldrich, St. Louis, MO, USA) released at a rate of 6 mg/kg/day in order to simulate the nicotine plasma level found in moderate to heavy smokers, or an equal volume of saline vehicle for the control^18^. Nicotine is released via the implanted osmotic pump throughout pregnancy and breastfeeding for 4 weeks from gestational day 6 to postnatal day 14.

### Slice preparation and RNA extraction

14 animals were randomly pooled together from different pregnant rats (n = 3). They were aged approximately 4 weeks (male and female), previously exposed to nicotine (n = 7) or saline (n = 7) through the placenta and after birth through breast milk were anesthetized with isoflurane before decapitation using a guillotine. Brains were rapidly removed and sectioned on a VT1200 semiautomatic vibrating blade microtome (Leica, Nussloch, Eisfeld, Germany) into 250 μm thick horizontal slices containing the top (PBP), middle (PIF), and bottom (PN) sub-regions of the VTA. Bilateral brain punches containing the VTA were collected under negative pressure using a 1 mm biopsy punch (Integra Miltex, VWR, Radnor, PA, USA) and deposited in 500 μL of ice-cold RNAlater (Invitrogen, Thermo Fisher Scientific, USA). Separate biopsy punches were used for each sub-region to prevent cross-contamination. Total RNA was isolated using RNeasy Mini Kit (Qiagen, Hilden, Germany) according to manufacturer’s instructions. Next, cDNA was prepared using High Capacity cDNA Reverse Transcription Kit (Applied Biosystems, Thermo Fisher Scientific, Carlsbad, CA, USA) according to manufacturer’s instructions and reverse transcription (RT) was performed on a T100 thermal cycler (Bio-Rad, Hercules, CA, USA).

### Real-time PCR

All primers used for all reactions were TaqMan Gene Expression Assays (Thermo Fisher Scientific, Carlsbad, CA, USA). GAD1 (Assay ID: Rn00690300-m1) and GAD2 (Assay ID: Rn00561244-m1) for GABA, Slc6a3 (Assay ID: Rn00562224-m1) and Th (Assay ID: Rn00562500-m1) for dopamine, and Slc17a6 (Assay ID: Rn00584780-m1) for dopamine and glutamate. The efficiency of mRNA primers was all initially tested in order to validate downstream results and determine the optimal amount of total RNA to use per reaction. Comparative Ct method (ΔΔCt) was also calculated using StepOnePlus Real-Time PCR System and used to determine relative expression values. Triplicate RT-qPCR reactions were performed in all validation experiments. Standard curves were created by step-wise 5x dilution of total RNA from midbrain samples from 20 ng to about 6 pg. Table 1 describes the mRNA primers and their corresponding validation results. Real-time PCR was carried out using TaqMan Fast Advanced Master Mix purchased from Applied Biosystems (Thermo Fisher Scientific, Carlsbad, CA, USA) and corresponding TaqMan Assays on a StepOnePlus Real-Time PCR System (Applied Biosystems, Thermo Fisher Scientific, Carlsbad, CA, USA) according to manufacturer’s instructions using the following parameters: 2 min at 50 °C, 2 min at 95 °C, 40 cycles at 1 sec at 95 °C and 20 sec at 60 °C. All reactions were performed in triplicate.

### Immunohistochemistry

The immunohistochemistry protocol with slight modifications was used according to Karadottir *et al*.^49^. The brain was cut at a thickness of 100 μm. Slices containing the VTA were collected in a 24 well plate and fixed in 4% PFA (Alfa Aesar, Haverhill, MA, USA) at 4 °C overnight. The next day, slices were washed 3 × 15 min in 0.1 M PBS. Slices were then transferred to a new 24 well plate containing 1 ml of blocking and permeabilizing solution which consists of 10% goat serum (life technologies, Thermo Fisher Scientific, USA), 0.5% Triton X-100, 0.05% NaN3, and 0.1 M PBS using a paint brush and placed in a black box on a shaker for 4 hours at room temperature. Primary anti-Tyrosine Hydroxylase (TH, AB152, MilliporeSigma, Burlington, MA, USA) was diluted at a 1:1000 ratio in 0.1 M PBS with 0.05% NaN3 and using a paint brush, slices were transferred to a new 24 well plate containing 500 of primary antibody dilution and incubated in the dark on a shaker at room temperature for 12–15 hours or overnight. Slices were washed the next day 4 times for 20 min in 0.1 M PBS. Application of the secondary antibody, Goat Anti-Rabbit IgG (ab150077, Abcam, Cambridge, MA, USA) at a dilution of 1:500 was done next and added to each well including the blank control for 4 hours in the dark on a shaker at room temperature. Finally, slices were washed 4 times for 20 min in 0.1 M PBS and mounted on microscope slides ready for imaging.

### Data analysis

All the statistical analyses were performed with GraphPad Prism 8.0 (GraphPad Software, Inc., San Diego, CA), including F test degrees of freedom. Statistical significance was assessed using repeated measures two-way analysis of variance (ANOVA) followed by Tukey’s post-hoc analysis. Unpaired t-tests with Welch’s correction were used when comparing gene expression profiles between nicotine and saline treatment groups in the sub-regions. Statistical information was given as t = t-value, p = p-value. Benjamini-Hochberg False Discovery Rate (FDR) p-values were calculated to correct for multiple testing^50^. Genes with FDR adjusted p < 0.05 were considered significantly differentially expressed. Sample sizes were calculated using standard power calculations, requiring an effect size of 30% at 80% power. Values are expressed as the arithmetic mean ± standard error of the mean (SEM). Throughout the manuscript, independent biological replicates are defined as independently performed experiments on material derived from different animals.
